# Sex Disparities After Coronary Artery Bypass Grafting and Hospital Quality

**DOI:** 10.1001/jamanetworkopen.2024.14354

**Published:** 2024-06-11

**Authors:** Catherine M. Wagner, Andrew M. Ibrahim

**Affiliations:** 1 National Clinician Scholar’s Program, Institute for Healthcare Policy and Innovation, University of Michigan, Ann Arbor; 2 Center for Healthcare Outcomes and Policy, University of Michigan, Ann Arbor; 3 Department of Surgery, Michigan Medicine, Ann Arbor

## Abstract

**Importance:**

Concern has been raised about persistent sex disparities after coronary artery bypass grafting, with female patients having higher mortality. However, whether these disparities persist across hospitals of different qualities is unknown.

**Objective:**

To evaluate sex disparities in 30-day mortality after coronary artery bypass grafting across high- and low-quality hospitals.

**Design, Setting, and Participants:**

This cross-sectional, retrospective cohort study evaluated Medicare beneficiaries undergoing coronary artery bypass grafting between October 1, 2015, and March 31, 2020. Data analysis was performed from July 1, 2023, to December 1, 2023.

**Exposures:**

The primary exposures were hospital quality and sex. For hospital quality, hospitals were placed into rank order by their overall risk-adjusted mortality rate and divided into quintiles.

**Main Outcome and Measures:**

Risk-adjusted 30-day mortality using a logistic regression model accounting for patient factors, including sex, age, comorbidities, elective vs unplanned admission, number of bypass grafts, use of arterial graft, and year of surgery.

**Results:**

A total of 444 855 beneficiaries (mean [SD] age, 71.5 [7.5] years; 120 333 [27.1%] female and 324 522 [72.9%] male) were studied. Compared with male beneficiaries, female beneficiaries were more likely to have an unplanned admission (66 425 [55.2%] vs 157 895 [48.7%], *P* < .001) and receive care at low-quality (vs high-quality) hospitals (odds ratio, 1.26; 95% CI, 1.23-1.29; *P* < .001). Overall, risk-adjusted female mortality was 4.24% (95% CI, 4.20%-4.27%), and male mortality was 2.75% (95% CI, 2.75%-2.77%), with an absolute difference of 1.48 (95% CI, 1.45-1.51) percentage points (*P* < .001). At the highest-quality hospitals, male mortality was 1.57% (95% CI, 1.56%-1.59%), and female mortality was 2.58% (95% CI, 2.54%-2.62%), with an absolute difference of 1.01 (95% CI, 0.97-1.04) percentage points (*P* < .001). At the lowest-quality hospitals, male mortality was 4.94% (95% CI, 4.88%-5.01%), and female mortality was 7.02% (95% CI, 6.90%-7.13%), with an absolute difference of 2.07 (95% CI, 1.95-2.19) percentage points (*P* < .001). Female beneficiaries receiving care at low-quality hospitals had a higher mortality than male beneficiaries receiving care at the high-quality hospitals (7.02% vs 1.57%, *P* < .001).

**Conclusions and Relevance:**

In this cohort study of Medicare beneficiaries undergoing coronary artery bypass grafting, female beneficiaries were more likely to receive care at low-quality hospitals, where the sex disparity in mortality was double that of high-quality hospitals. Quality improvement targeting low-quality hospitals as well as equitable referral of female beneficiaries to higher-quality hospitals may narrow the sex disparity after coronary artery bypass grafting.

## Key Points

**Question:**

Is the sex outcomes disparity for Medicare beneficiaries undergoing high-risk surgery associated with the quality of hospital where beneficiaries receive care?

**Findings:**

In this retrospective cohort study of 444 855 Medicare beneficiaries undergoing coronary artery bypass grafting, female patients undergoing coronary artery bypass grafting were more likely to receive care at low-quality hospitals where the sex disparity in mortality doubled that of high-quality hospitals.

**Meaning:**

Quality improvement targeting low-quality hospitals as well as equitable referral of female beneficiaries to higher-quality hospitals may narrow the sex disparity after coronary artery bypass grafting.

## Teaser

This cohort study of Medicare beneficiaries evaluates the association between hospital quality and sex disparities in mortality after coronary artery bypass grafting.

## Introduction

Health equity has long been identified as a priority from leading government agencies and health care societies.^1,2,3^ Increasingly, there have been national calls for greater focus on sex disparities, including efforts through the National Institutes of Health requiring the inclusion of both sexes in basic science and clinical trials to combat historic underinclusion of female patients in research and recent legislation from the White House that specifically prioritizes research in women’s health (The White House Initiative on Women’s Health Research).^4,5^ To eliminate disparities, government agencies have identified improving access to high-quality care as a possible policy lever.^2^

However, the association of hospital quality with sex disparities is not well understood. A particular blind spot exists with high-risk surgery, such as coronary artery bypass grafting, for which female patients have had significantly higher mortality than male patients for decades.^6,7,8,9^ On one hand, the sex disparity may be consistent regardless of hospital quality and more attributable to patient-specific risk factors.^10,11^ Alternatively, the sex disparity may widen as hospital quality worsens. If hospital quality contributes to worse outcomes in female patients, policy could be enacted to have targeted quality improvement at low-quality hospitals or more equitably refer female patients to higher-quality hospitals to narrow the disparity.^12^

In that context, we designed a national study of Medicare beneficiaries undergoing high-risk surgery to evaluate the association of sex disparities with hospital quality. In particular, we sought to stratify hospitals into quintiles of quality to compare where patients receive care and evaluate whether outcome disparities changed across hospital quality.

## Methods

### Data Source and Cohort Selection

All Medicare beneficiaries undergoing coronary artery bypass grafting from October 1, 2015, to March 31, 2020, were identified using 100% claims data from the Medicare Provider Analysis and Review (MEDPAR) file, including fee-for-service and Medicare Advantage beneficiaries. Medicare beneficiaries with at least 3 months of continuous enrollment before surgery and 12 months of enrollment after surgery were included. Coronary artery bypass grafting was selected because it is one of the most common high-risk operations performed nationally and there are known, persistent sex disparities.^6,7^ Moreover, coronary artery bypass grafting has also been a model high-risk procedure that health care systems and regulatory health agencies (ie, Centers for Medicare & Medicaid Services [CMS]) continue to target for quality improvement.^13^ Procedure codes from the *International Statistical Classification of Diseases and Related Health Problems, Tenth Revision* (*ICD-10*) were used to define the cohort (eTable 1 in Supplement 1). Patient demographics and comorbidities were collected. In Medicare, race and ethnicity are identified through self-report, and categories include Asian, Black, Hispanic, North American Native, White, and other. Race and ethnicity are included in this study as a social construct and not as a biological risk factor. Data on race and ethnicity were collected to evaluate how intersectional identities (eg, Black female vs White male) may affect mortality. Hospital characteristics, including hospital size, nurse to patient ratio, and teaching status, were linked to the MEDPAR file with unique hospital identifiers from the American Hospital Association annual survey.^14^ Among the data presented in this study, the only variable with missingness was race (n = 7609 [1.8%]), which was labeled as “unknown.” Because race is not included in this risk adjustment, all identified beneficiaries were included. This study was determined to be exempt from review by the University of Michigan Institutional Review Board because it was no more than minimal risk. Patient consent was waived by the institutional review board because all data were deidentified. This study follows the Strengthening the Reporting of Observational Studies in Epidemiology (STROBE) reporting guideline.

### Outcome

The primary outcome in this study was 30-day mortality, which included in-hospital mortality and death within 30 days postoperatively. In-hospital mortality was identified with vital status at time of discharge. Deaths occurring within 30 days of the index operation but outside the hospital were identified using the Medicare Denominator File. All follow-up for 30-day mortality was 100% complete.

### Hospital Quality

Hospital quality was defined by placing hospitals into quintiles by their risk-adjusted rate of 30-day mortality. Hospitals in the quintile with the lowest 30-day mortality were characterized as highest quality, and hospitals in the quintile with the highest 30-day mortality were characterized as lowest quality.

The study used 30-day mortality rates to determine hospital quality for several reasons. First, it is an established approach used in several prior studies evaluating surgical quality at the hospital level.^12,15,16,17,18,19^ Second, coronary artery bypass grafting mortality is a core measure in national hospital quality ratings used by the CMS.^20^ Third, 30-day mortality after coronary artery bypass grafting is validated by the National Quality Forum and other leading national organizations (ie, Society of Thoracic Surgeons).^21,22^ Fourth, other quality measures, such as the CMS star ratings, have significant missingness and miss 20% to 26% of hospitals nationally.^23^ Given this, we used each hospital’s risk-adjusted coronary artery bypass graft mortality because it is a validated quality measure and would be inclusive of all patients in our dataset.

### Statistical Analysis

The overall goal of this analysis was to evaluate the association between hospital quality and sex disparities among patients undergoing coronary artery bypass grafting. First, patient demographics were compared by sex. Continuous variables were normally distributed and are presented as mean (SD) and compared with a 2-tailed *t* test. Categorical variables are presented as number (percentage) and compared using the χ^2^ test.

Second, the quintiles of hospital quality were established. To do this, a 2-tier hierarchical logistic regression was performed to calculate risk-adjusted 30-day mortality, accounting for variation in patient population across different hospitals. With the use of a well-established risk-adjustment model, a cross-sectional comparison of the entire cohort was performed using a logistic regression adjusting for age, 27 comorbidities, surgery year, admission type (elective vs unplanned), number of coronary artery bypass grafts, and use of arterial grafts.^24,25,26,27^ Surgery year was included as a categorical model to account for secular trends. Use of an arterial graft was determined using *ICD-10* codes (eTable 2 in Supplement 1). The number of bypass grafts indicated by each *ICD-10* code was summed across the first 25 procedure codes to determine the total number of bypass grafts performed. Patient sex was interacted with the terms of the model, allowing the coefficients to vary by sex, to account for the different ways male and female patients experience the health care system. Hospital characteristics were not included in the model because hospital was part of the exposure, although the coefficient of sex was allowed to vary at the hospital level. Robust SEs were applied to all outcome estimates to account for clustering of patients within hospitals.

After identification of quintiles of hospital quality, hospital characteristics were compared across the different quintiles. Continuous variables were tested for normality using the Shapiro-Wilk test. Categorial variables are presented as number (percentage), normally distributed variables are presented as mean (SD), and skewed variables are presented as median (IQR). Hospital characteristics were compared using the χ^2^ test, 2-tailed *t* test, or Wilcoxon rank sum test as appropriate. Finally, differences in 30-day mortality by sex were described across high- and low-quality hospitals.

Several sensitivity analyses were performed to test the robustness of our findings. First, hospital quality was also determined by mortality rates of male beneficiaries only to address the potential endogeneity of defining hospital quality by postoperative mortality and comparing mortality rates by sex. Second, the percentage of female patients at each hospital was included in the model to account for different patient populations treated, which may influence overall hospital mortality. Data analysis was performed from July 1, 2023, to December 1, 2023. All analyses were performed using Stata MP software, version 16.1 (StatCorp LLC). All hypothesis tests were 2-sided, and a significance level of *P* < .05 was used.

## Results

### Patient and Hospital Characteristics

Of 444 855 beneficiaries included, 324 522 (72.9%) were male and 120 333 (27.1%) were female. The mean (SD) age for both male and female patients was 71.5 (7.5) years (Table 1). Of the female beneficiaries, 2165 (1.8%) were Asian, 13 672 (11.4%) Black, 2537 (2.1%) Hispanic, 838 (0.7%) North American Native, 98 165 (81.6%) White, 1995 (1.7%) other, and 961 (0.8%) of unknown race and ethnicity. Of the male beneficiaries, 5703 (1.8%) were Asian, 18 284 (5.3%) Black, 6087 (1.9%) Hispanic, 1637 (0.5%) North American Native, 278 628 (85.9%) White, 7275 (2.2%) other, and 6908 (2.1%) of unknown race and ethnicity. Additionally, female beneficiaries had more comorbidities than male beneficiaries (≥2 Elixhauser comorbidities: 110 254 [91.6%] vs 277 721 [85.6%], *P* < .001) and were more likely to have an unplanned surgery (66 425 [55.2%] vs 157 895 [48.7%], *P* < .001). Operatively, female beneficiaries received fewer bypass grafts than male beneficiaries and were less likely to receive an arterial conduit.

**Table 1.** Patient Demographics by Sex

Characteristic	No. (%)		*P* value
	Male (n = 324 522)	Female (n = 120 333)	
Age, mean (SD), y	71.5 (7.4)	71.5 (7.9)	.02
Race^a^			
Asian	5703 (1.8)	2165 (1.8)	<.001
Black	18 284 (5.3)	13 672 (11.4)	
Hispanic	6087 (1.9)	2537 (2.1)	
North American Native	1637 (0.5)	838 (0.7)	
White	278 628(85.9)	98 165 (81.6)	
Other	7275 (2.2)	1995 (1.7)	
Unknown	6908 (2.1)	961 (0.8)	
Selected comorbidities			
Hypertension	221 031 (68.1)	80 174 (66.6)	<.001
Diabetes	155 062 (48.0)	66 025 (54.9)	<.001
Chronic pulmonary disease	73 088 (22.5)	33 576 (27.9)	<.001
No. of Elixhauser comorbidities^b^			
0	8390 (2.6)	1539 (1.3)	<.001
1	38 411 (11.8)	8540 (7.1)	
≥2	277 721 (85.6)	110 254 (91.6)	
Admission type			
Unplanned (urgent or emergency) surgery	157 895 (48.7)	66 425 (55.2)	<.001
No. of bypass grafts			
1	16 867 (5.2)	9274 (7.7)	<.001
2	56 051 (17.3)	27 533 (22.9)	
3	130 485 (40.2)	50 838 (42.3)	
≥4	121 119 (37.3)	32 688 (27.2)	
Arterial conduit	294 165 (90.7)	106 659 (88.6)	<.001

^a^ Race and ethnicity are self-reported in Medicare, and beneficiaries may select only 1 option from the provided list. Unknown indicates beneficiaries for whom race information is missing. In this study, race and ethnicity are included as a social construct and not a biological risk factor.

^b^ Elixhauser comorbidities are a set of 30 comorbidities present on admission that are identified in claims data and are known to be associated with outcomes.^24^ Use of Elixhauser comorbidities is a well-established risk-adjustment method in Medicare claims.^28,29,30^

### Hospital Characteristics

Hospital characteristics differed across the quintiles of hospital quality. The lowest-quality hospitals were more likely to be for-profit hospitals and had the lowest nurse to patient ratio, the smallest hospital bed size, and the lowest coronary artery bypass grafting volume per hospital (Table 2). Although fewer patients went to low-quality hospitals than high-quality hospitals, the lowest-quality hospitals had the highest proportion of female beneficiaries (Table 3). Female beneficiaries had 26% higher odds of receiving care at the lowest-quality hospitals vs the highest-quality hospitals (odds ratio, 1.26; 95% CI, 1.23-1.29; *P* < .001).

**Table 2.** Hospital Characteristics by Hospital Quality^a^

Characteristic	Highest quality (n = 245)	High quality (n = 244)	Average quality (n = 244)	Low quality (n = 244)	Lowest quality (n = 244)	*P* value
Profit status, No. (%)						
For profit	32 (13.1)	33 (13.5)	41 (16.8)	52 (21.3)	89 (36.5)	<.001
Not for profit	188 (76.7)	189 (77.5)	179 (73.4)	172 (70.5)	124 (50.8)	
Other	25 (10.2)	22 (9.0)	24 (9.8)	20 (8.2)	31 (12.7)	
Teaching hospital	204 (83.3)	216 (88.5)	212 (86.8)	213 (87.3)	198 (81.1)	.14
Nurse to patient ratio, median (IQR)	9 (7-11)	9 (8-11)	9 (8-10)	8 (7-10)	8 (6-9)	<.001
Hospital region						
Northeast	46 (18.8)	39 (16.0)	34 (13.9)	25 (10.2)	12 (4.9)	<.001
West	67 (27.3)	47 (19.3)	48 (19.8)	44 (18.0)	57 (23.4)	.08
Midwest	64 (26.1)	74 (30.3)	59 (24.2)	72 (29.5)	51 (20.9)	.11
South	77 (31.4)	84 (34.4)	103 (42.2)	103 (42.2)	124 (50.8)	<.001
No. of hospital beds, median (IQR)	305 (195-449)	337 (227-525)	342 (211-516)	313 (219-443)	282 (194-406)	.002
Coronary artery bypass grafting volume, median (IQR)	344 (146-582)	336 (193-580)	291 (139-538)	257 (138-471)	178 (64-321)	<.001

^a^ First, hospital-level risk-adjusted 30-day mortality was calculated. Second, hospitals were placed in rank order by 30-day mortality divided into quintiles of quality. The quintile with the lowest 30-day mortality was deemed “highest quality,” and the quintile with the highest 30-day mortality was deemed “lowest quality.” Risk adjustment included patient characteristics (ie, age, sex, and comorbidities) and operative characteristics (ie, number of bypass grafts).

**Table 3.** Comparison of the Percentage of Female Beneficiaries and the Absolute Difference in Risk-Adjusted Mortality Between Male and Female Beneficiaries by Hospital Quality^a^

Hospital quality	Female beneficiaries, No. (%)	Absolute difference in risk-adjusted mortality (95% CI), percentage points
Highest quality (n = 106 718)	27 060 (24.9)	1.01 (0.97-1.04)
High quality (n = 108 433)	28 699 (26.2)	1.21 (1.16-1.26)
Average quality (n = 90 403)	24 697 (27.3)	1.37 (1.32-1.43)
Low quality (n = 82 734)	23 415 (28.6)	1.59 (1.52-1.67)
Lowest quality (n = 56 567)	16 462 (29.5)	2.07 (1.95-2.19)

^a^ First, hospital-level risk-adjusted 30-day mortality was calculated. Second, hospitals were placed in rank order by 30-day mortality divided into quintiles of quality. The quintile with the lowest 30-day mortality was deemed “highest quality,” and the quintile with the highest 30-day mortality was deemed “lowest quality.” Risk adjustment included patient characteristics (ie, age, sex, and comorbidities) and operative characteristics (ie, number of bypass grafts). The absolute difference was calculated by subtracting male beneficiaries’ risk-adjusted rate of 30-day mortality from female beneficiaries’ risk-adjusted rate of 30-day mortality (ie, female – male). The difference in distribution of female beneficiaries across hospital quality was significant (*P* < .001). The difference in mortality by sex was significant across all levels of hospital quality (*P* < .001).

### Interaction Among Hospital Quality, Sex, and 30-Day Mortality

**Figure 1.**
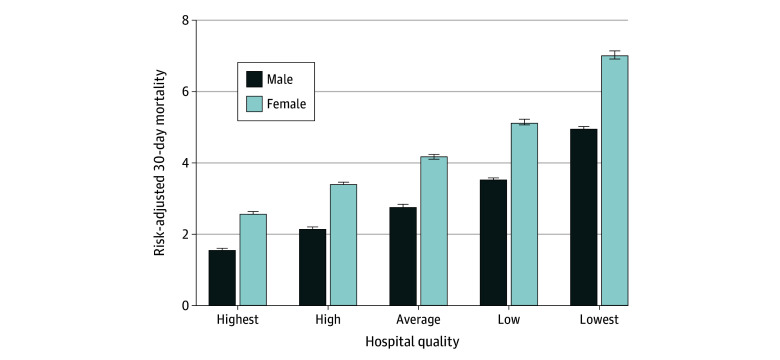
Risk Adjusted 30-Day Mortality Stratified by Sex and Hospital Quality First, hospital-level risk-adjusted 30-day mortality was calculated. Second, hospitals were placed in rank order by 30-day mortality divided into quintiles of quality. The quintile with the lowest 30-day mortality was deemed “highest quality” and the quintile with the highest 30-day mortality was deemed “lowest quality.” Risk adjustment included patient characteristics (ie, age, sex, and comorbidities) and operative characteristics (ie, number of bypass grafts). The differences by sex and hospital quality were statistically significant (*P* < .001). Error bars indicate 95% CIs.

**Figure 2.**
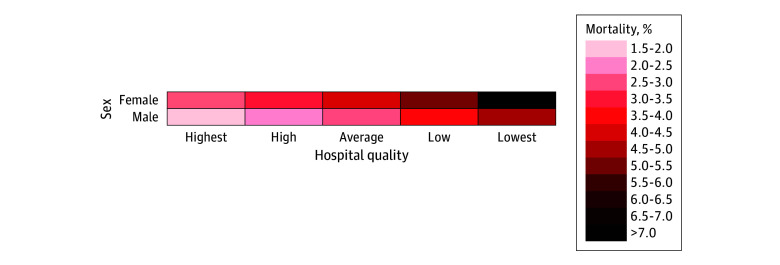
Risk Matrix of Risk-Adjusted 30-Day Mortality by Sex and Hospital Quality In the risk matrix, as a square gets darker, the risk-adjusted mortality is higher. Female beneficiaries at low-quality hospitals had a greater than 4-fold higher mortality than male beneficiaries at high-quality hospitals.

Overall, risk-adjusted female mortality was 4.24% (95% CI, 4.20%-4.27%), and male mortality was 2.75% (95% CI, 2.75%-2.77%), with an absolute difference of 1.48 (95% CI, 1.45-1.51) percentage points (*P* < .001). The sex disparity in mortality increased as hospital quality worsened. At the highest-quality hospitals, male mortality was 1.57% (95% CI, 1.56%-1.59%), and female mortality was 2.58% (95% CI, 2.54%-2.62%), with an absolute difference of 1.01 (95% CI, 0.97-1.04) percentage points (*P* < .001) (Figure 1). At the lowest-quality hospitals, male mortality was 4.94% (95% CI, 4.88%-5.01%), and female mortality was 7.02% (95% CI, 6.90%-7.13%), with an absolute difference of 2.07 (95% CI, 1.95-2.19) percentage points (*P* < .001). In other words, female beneficiaries receiving care at the lowest-quality hospitals had a greater than 4-fold higher mortality than male beneficiaries receiving care at the highest-quality hospitals (7.02% vs 1.57%) (Figure 2).

The results of the sensitivity analyses mirror our main findings. These data are provided in eTables 3 and 4 in Supplement 1.

## Discussion

In this cohort study of Medicare beneficiaries undergoing coronary artery bypass grafting from 2015 to 2020, female beneficiaries were more likely to receive care at low-quality hospitals, and the sex disparity in 30-day mortality increased as hospital quality worsened. There was a doubling in the disparity from high-quality to low-quality hospitals. Taken together, our findings suggest that policy focused on quality improvement for low-quality hospitals and more equitable referral of female beneficiaries to high-quality hospitals may narrow sex mortality disparities after high-risk surgery.

Prior work has found that hospital quality contributes to outcomes disparities. Several groups have found that Black patients are more likely to undergo surgery at low-quality hospitals with higher postoperative mortality.^12,28,31,32^ Similarly, recent work found that Medicare beneficiaries from areas with highest neighborhood deprivation were more likely to undergo surgery at the lowest-quality hospitals.^29^ Moreover, beneficiaries from the highest deprivation areas who received care at the lowest-quality hospitals had nearly triple the mortality of beneficiaries from the lowest deprivation areas who received care at the highest-quality hospitals.^29^ Our work builds on these important studies by demonstrating how hospital quality impacts sex outcomes disparities. Although there have been recent calls to action to resolve sex outcome disparities after high-risk surgery,^33,34^ the role of hospital quality on sex disparities has not been examined before in a large, nationally representative cohort.

Although the mechanisms underlying the persistent sex disparities are difficult to elucidate, our findings point toward multiple plausible mechanisms. First, female beneficiaries are a higher-risk population than male beneficiaries (eFigure in Supplement 1). In this study, female beneficiaries were more likely to have an unplanned (vs elective) admission and had a higher comorbidity burden than male beneficiaries, supporting prior findings^35,36^ that female beneficiaries with coronary artery disease are referred later for surgery. This later disease presentation may be due to understudied biological differences driving female patients’ later presentation with coronary artery disease but also reflects that ischemic heart disease in female patients is underdiagnosed and undertreated.^35,36^ Moreover, once female patients are diagnosed with ischemic heart disease, they are less likely to receive guideline-directed medical therapy and have longer delays in time to revascularization.^37^ Together, these factors contribute to higher-acuity presentation in female patients. High-quality hospitals may manage these risk factors more effectively and have more resources available to provide state-of-the-art care to this higher-risk population.^38,39^

Hospital annual procedural volume may also contribute to the disparity in outcomes among male and female patients. Prior work in cardiac procedures has demonstrated a clear volume-outcomes relationship, with higher-volume centers demonstrating better surgical outcomes.^40,41^ In the current study, female patients were more likely to undergo their procedures at lower-volume hospitals and were less likely to receive arterial grafting, which is a more technically challenging revascularization strategy but associated with superior outcomes.^42,43^ Specialty care at a high-volume hospital may be particularly relevant for female patients, who often have smaller anatomy, making coronary artery bypass grafting more difficult.^42,44^ Indeed, it is probably a synergistic combination of both patient risk and hospital experience that leads to the outcome differences by sex across quality. High-quality hospitals may be better equipped to care for female patients, who are a higher-risk and more technically challenging patient population than their male counterparts.

Our findings have several policy implications. First, equitable referral of female patients to high-quality hospitals may narrow sex outcome disparities after high-risk surgery. The rationale underscoring this recommendation is seen both in our data as well as in national calls for female-specific centers of excellence for coronary artery bypass grafting.^33,34^ Building on this, in light of evidence that surgeon and patient sex concordance improves outcomes specifically for female patients, practitioner-level policies aimed at improving gender diversity in cardiothoracic surgery, where more than 90% of practicing cardiothoracic surgeons are male, could narrow this disparity.^45,46^ Second, as is apparent from the geographic heterogeneity in hospital quality in this study, where high-quality hospitals were concentrated in the North and low-quality hospitals were concentrated in the South, access to high-quality hospitals is not uniform, and equitable referral alone cannot resolve the sex disparity. In conjunction with referral patterns, targeted improvement for low-quality hospitals may be a needed strategy to eliminate sex-based disparities after surgery.^12^ Doing so, however, would first require a uniform commitment of hospitals to measure sex disparities in high-risk groups.^47^ Currently, there are no such requirements from payers or policymakers at the national level. Third, because there is increasing national interest in sex outcomes disparities and leveraging hospital quality to narrow those disparities, our findings are timely.^2,3,4^ Policy could specifically target sex disparities as a quality measure or incentivize equitable referral to higher-quality centers.

### Limitations

This work has several limitations. First, this study uses administrative claims data, which lack the clinical granularity of registry data. To mitigate this, we chose a primary outcome, 30-day mortality, that can be identified in claims data as accurately as registry data.^7,48^ Additionally, the sex disparity after coronary artery bypass grafting has been described using both claims data and clinical granular registry data (ie, Society of Thoracic Surgeons database) and is pervasive regardless of the dataset used, so this is not a unique finding to this claims-based analysis. Therefore, it seems unlikely that the findings of this study (exploring the sex disparity as a function of hospital quality) are entirely attributable to the use of claims data, although replication in a more clinically granular dataset would be informative. Second, only Medicare beneficiaries who were 65 years or older were included in this study, which may limit generalizability to other populations undergoing coronary artery bypass grafting. However, most patients who undergo coronary artery bypass grafting are older than 65 years and qualify for Medicare; thus, they are represented in this study. Although the sex disparity after coronary artery bypass grafting is pervasive regardless of age, in some work, the sex disparity is magnified in younger populations, and understanding the role of hospital quality could help to achieve equity for all age groups.^5,8,42^ Finally, 30-day mortality after coronary artery bypass grafting may not be an accurate representative for hospital quality. However, 30-day mortality after coronary artery bypass grafting is a validated quality measure by the National Quality Foundation and is used as a quality measure by national cardiac surgery societies and national hospital ranking methods. Additionally, 30-day mortality is commonly used in surgical quality research.^12,17,18,19,20,21,30^ Future work could examine other markers of hospital quality to further explore how hospital-level factors contribute to the sex disparity after coronary artery bypass grafting.

## Conclusions

In this cohort study of Medicare beneficiaries undergoing coronary artery bypass grafting, female beneficiaries had higher 30-day mortality than male beneficiaries, a disparity that increased as hospital quality worsened. Additionally, female beneficiaries were more concentrated at low-quality centers. Policy aimed at equitable referral of female patients to high-quality centers and targeted improvement of low-quality hospitals may narrow these disparities.

## Notes

Supplement 1. **eTable 1.** International Classification of Diseases Tenth Revision (ICD-10) Codes Used to Define the Cohort **eTable 2.** International Classification of Diseases Tenth Revision (ICD-10) Codes Used to Identify Arterial Grafting **eTable 3.** Definition of Hospital Quality Based Only on Risk-Adjusted Male Mortality **eTable 4.** Definition of Hospital Quality With Risk-Adjustment Controlling for Percentage of Female Patients at the Hospital Level **eFigure.** Conceptual Model Evaluating Factors That Contribute to Females’ Higher Mortality After Coronary Artery Bypass Grafting

Supplement 2.
Data Sharing Statement

## References

[1] US Department of Health and Human Services. Agency Equity Action Plan. 2023. Accessed April 23, 2024. https://www.hhs.gov/sites/default/files/hhs-equity-action-plan.pdf

[2] McIver DL. CMS Framework for Health Equity 2022–2032. April 2022. Accessed April 24, 2024. https://www.cms.gov/files/document/cms-framework-health-equity.pdf

[3] Centers for Medicare & Medicaid Services. CMS Framework for Health Equity 2022-2023. Accessed August 31, 2023. https://www.cms.gov/files/document/cms-framework-health-equity-2022.pdf

[4] The White House. Launch of White House initiative on women’s health research. Published November 17, 2023. Accessed November 29, 2023. https://www.whitehouse.gov/gpc/briefing-room/2023/11/17/launch-of-white-house-initiative-on-womens-health-research/

[5] Arnegard ME, Whitten LA, Hunter C, Clayton JA. Sex as a biological variable: a 5-year progress report and call to action. J Womens Health (Larchmt). 2020;29(6):858-864. doi:10.1089/jwh.2019.8247 31971851 PMC7476377

[6] Gaudino M, Chadow D, Rahouma M, . Operative outcomes of women undergoing coronary artery bypass surgery in the US, 2011 to 2020. JAMA Surg. 2023;158(5):494-502. doi:10.1001/jamasurg.2022.8156 36857059 PMC9979009

[7] Angraal S, Khera R, Wang Y, . Sex and race differences in the utilization and outcomes of coronary artery bypass grafting among Medicare beneficiaries, 1999-2014. J Am Heart Assoc. 2018;7(14):e009014. doi:10.1161/JAHA.118.009014 30005557 PMC6064835

[8] Enumah ZO, Canner JK, Alejo D, . Persistent racial and sex disparities in outcomes after coronary artery bypass surgery: a retrospective clinical registry review in the drug-eluting stent era. Ann Surg. 2020;272(4):660-667. doi:10.1097/SLA.0000000000004335 32932322 PMC8491278

[9] Vaccarino V, Abramson JL, Veledar E, Weintraub WS. Sex differences in hospital mortality after coronary artery bypass surgery: evidence for a higher mortality in younger women. Circulation. 2002;105(10):1176-1181. doi:10.1161/hc1002.105133 11889010

[10] Swaminathan RV, Feldman DN, Pashun RA, . Gender differences in in-hospital outcomes after coronary artery bypass grafting. Am J Cardiol. 2016;118(3):362-368. doi:10.1016/j.amjcard.2016.05.004 27269585

[11] Wang J, Yu W, Zhao D, Liu N, Yu Y. In-hospital and long-term mortality in 35,173 Chinese patients undergoing coronary artery bypass grafting in Beijing: impact of sex, age, myocardial infarction, and cardiopulmonary bypass. J Cardiothorac Vasc Anesth. 2017;31(1):26-31. doi:10.1053/j.jvca.2016.08.004 27771273

[12] Dimick J, Ruhter J, Sarrazin MV, Birkmeyer JD. Black patients more likely than whites to undergo surgery at low-quality hospitals in segregated regions. Health Aff (Millwood). 2013;32(6):1046-1053. doi:10.1377/hlthaff.2011.1365 23733978 PMC4789147

[13] Centers for Medicare & Medicaid Services. Outcome and payment measures. September 6, 2023. Accessed August 31, 2023. https://www.cms.gov/medicare/quality-initiatives-patient-assessment-instruments/hospitalqualityinits/outcomeandpaymentmeasures

[14] American Hospital Association. AHA annual survey database. Accessed December 18, 2023. https://www.ahadata.com/aha-annual-survey-database

[15] Osborne NH, Upchurch GR Jr, Mathur AK, Dimick JB. Explaining racial disparities in mortality after abdominal aortic aneurysm repair. J Vasc Surg. 2009;50(4):709-713. doi:10.1016/j.jvs.2009.05.020 19703760

[16] Rothenberg BM, Pearson T, Zwanziger J, Mukamel D. Explaining disparities in access to high-quality cardiac surgeons. Ann Thorac Surg. 2004;78(1):18-24. doi:10.1016/j.athoracsur.2004.01.021 15223394

[17] Ghaferi AA, Birkmeyer JD, Dimick JB. Variation in hospital mortality associated with inpatient surgery. N Engl J Med. 2009;361(14):1368-1375. doi:10.1056/NEJMsa0903048 19797283

[18] Glance LG, Thirukumaran CP, Li Y, Gao S, Dick AW. Improving the accuracy of hospital quality ratings by focusing on the association between volume and outcome. Health Aff (Millwood). 2020;39(5):862-870. doi:10.1377/hlthaff.2019.00778 32364861 PMC7423250

[19] Birkmeyer JD, Siewers AE, Finlayson EVA, . Hospital volume and surgical mortality in the United States. N Engl J Med. 2002;346(15):1128-1137. doi:10.1056/NEJMsa012337 11948273

[20] Centers for Medicare & Medicaid Services. Hospitals: overall hospital quality star rating. Accessed August 31, 2023. https://data.cms.gov/provider-data/topics/hospitals/overall-hospital-quality-star-rating/#measure-included-by-categories

[21] Society of Thoracic Surgeons. Performance measures. Accessed December 23, 2022. https://www.sts.org/quality-safety/performance-measures

[22] Google. nqf 2558. Accessed August 31, 2023. https://www.google.com/search?q=nqf+2558&rlz=1C5CHFA_enUS852US855&oq=nqf+2558&aqs=chrome.69i57j69i60.1610j0j4&sourceid=chrome&ie=UTF-8

[23] Yale New Haven Health Services Corporation/Center for Outcomes Research and Evaluation. Overall Hospital Quality Star Rating on Care Compare Methodology Report (v4.0). January 2021. Accessed April 30, 2024. https://qualitynet.cms.gov/files/603966dda413b400224ddf50?filename=Star_Rtngs_CompMthdlgy_v4.1.pdf

[24] Elixhauser A, Steiner C, Harris DR, Coffey RM. Comorbidity measures for use with administrative data. Med Care. 1998;36(1):8-27. doi:10.1097/00005650-199801000-00004 9431328

[25] Southern DA, Quan H, Ghali WA. Comparison of the Elixhauser and Charlson/Deyo methods of comorbidity measurement in administrative data. Med Care. 2004;42(4):355-360. doi:10.1097/01.mlr.0000118861.56848.ee 15076812

[26] Iezzoni LI, Daley J, Heeren T, . Identifying complications of care using administrative data. Med Care. 1994;32(7):700-715. doi:10.1097/00005650-199407000-00004 8028405

[27] Rathore SS, Epstein AJ, Volpp KGM, Krumholz HM. Hospital coronary artery bypass graft surgery volume and patient mortality, 1998-2000. Ann Surg. 2004;239(1):110-117. doi:10.1097/01.sla.0000103066.22732.b8 14685108 PMC1356200

[28] Bonner SN, Kunnath N, Dimick JB, Ibrahim AM. Hospital-level racial and ethnic segregation among Medicare beneficiaries undergoing common surgical procedures. JAMA Surg. 2022;157(10):961-964. doi:10.1001/jamasurg.2022.3135 35921121 PMC9350841

[29] Diaz A, Lindau ST, Obeng-Gyasi S, Dimick JB, Scott JW, Ibrahim AM. Association of hospital quality and neighborhood deprivation with mortality after inpatient surgery among Medicare beneficiaries. JAMA Netw Open. 2023;6(1):e2253620. doi:10.1001/jamanetworkopen.2022.53620 36716028 PMC9887494

[30] Ibrahim AM, Hughes TG, Thumma JR, Dimick JB. Association of hospital critical access status with surgical outcomes and expenditures among Medicare beneficiaries. JAMA. 2016;315(19):2095-2103. doi:10.1001/jama.2016.5618 27187302

[31] Epstein AJ, Gray BH, Schlesinger M. Racial and ethnic differences in the use of high-volume hospitals and surgeons. Arch Surg. 2010;145(2):179-186. doi:10.1001/archsurg.2009.268 20157087

[32] Rangrass G, Ghaferi AA, Dimick JB. Explaining racial disparities in outcomes after cardiac surgery: the role of hospital quality. JAMA Surg. 2014;149(3):223-227. doi:10.1001/jamasurg.2013.4041 24402245

[33] Zwischenberger BA, Lawton JS. A call to action to improve outcomes in women undergoing surgical coronary revascularization. JAMA Surg. 2023;158(5):502-503. doi:10.1001/jamasurg.2022.8163 36857043

[34] Zwischenberger BA, Jawitz OK, Lawton JS. Coronary surgery in women: how can we improve outcomes. JTCVS Tech. 2021;10:122-128. doi:10.1016/j.xjtc.2021.09.051 34977714 PMC8691860

[35] Hyun KK, Redfern J, Patel A, . Gender inequalities in cardiovascular risk factor assessment and management in primary healthcare. Heart. 2017;103(7):492-498. doi:10.1136/heartjnl-2016-310216 28249996

[36] Aggarwal NR, Patel HN, Mehta LS, . Sex differences in ischemic heart disease: advances, obstacles, and next steps. Circ Cardiovasc Qual Outcomes. 2018;11(2):e004437. doi:10.1161/CIRCOUTCOMES.117.004437 29449443

[37] Roswell RO, Kunkes J, Chen AY, . Impact of sex and contact-to-device time on clinical outcomes in acute ST-segment elevation myocardial infarction—findings from the National Cardiovascular Data Registry. J Am Heart Assoc. 2017;6(1):e004521. doi:10.1161/JAHA.116.004521 28077385 PMC5523636

[38] Wakeam E, Hevelone ND, Maine R, . Failure to rescue in safety-net hospitals: availability of hospital resources and differences in performance. JAMA Surg. 2014;149(3):229-235. doi:10.1001/jamasurg.2013.3566 24430015

[39] Ghaferi AA, Osborne NH, Birkmeyer JD, Dimick JB. Hospital characteristics associated with failure to rescue from complications after pancreatectomy. J Am Coll Surg. 2010;211(3):325-330. doi:10.1016/j.jamcollsurg.2010.04.025 20800188

[40] Nallamothu BK, Saint S, Ramsey SD, Hofer TP, Vijan S, Eagle KA. The role of hospital volume in coronary artery bypass grafting: is more always better? J Am Coll Cardiol. 2001;38(7):1923-1930. doi:10.1016/S0735-1097(01)01647-3 11738295

[41] Gonzalez AA, Dimick JB, Birkmeyer JD, Ghaferi AA. Understanding the volume-outcome effect in cardiovascular surgery: the role of failure to rescue. JAMA Surg. 2014;149(2):119-123. doi:10.1001/jamasurg.2013.3649 24336902 PMC4016988

[42] Jawitz OK, Lawton JS, Thibault D, . Sex differences in coronary artery bypass grafting techniques: a Society of Thoracic Surgeons Database analysis. Ann Thorac Surg. 2022;113(6):1979-1988. doi:10.1016/j.athoracsur.2021.06.039 34280377

[43] Heidenreich PA, Bozkurt B, Aguilar D, . 2021 ACC/AHA/SCAI Guideline for Coronary Artery Revascularization: a report of the American College of Cardiology/American Heart Association Joint Committee on Clinical Practice Guidelines. Circulation. 2022;145(18):e895-e1032. 35363499 10.1161/CIR.0000000000001063

[44] Lawton JS, Barner HB, Bailey MS, . Radial artery grafts in women: utilization and results. Ann Thorac Surg. 2005;80(2):559-563. doi:10.1016/j.athoracsur.2005.02.055 16039204

[45] Wallis CJD, Jerath A, Coburn N, . Association of surgeon-patient sex concordance with postoperative outcomes. JAMA Surg. 2022;157(2):146-156. doi:10.1001/jamasurg.2021.6339 34878511 PMC8655669

[46] Association of American Medical Colleges. Active Physicians by Sex and Specialty. 2021. Accessed March 7, 2024. https://www.aamc.org/data-reports/workforce/data/active-physicians-sex-specialty-2021

[47] Frist WH. Overcoming disparities in U.S. health care. Health Aff (Millwood). 2005;24(2):445-451. doi:10.1377/hlthaff.24.2.445 15757929

[48] Birman-Deych E, Waterman AD, Yan Y, Nilasena DS, Radford MJ, Gage BF. Accuracy of *ICD-9-CM* codes for identifying cardiovascular and stroke risk factors. Med Care. 2005;43(5):480-485. doi:10.1097/01.mlr.0000160417.39497.a9 15838413

