# Supplementary material for: Sex Disparities After Coronary Artery Bypass Grafting and Hospital Quality
Source: JAMA Netw Open. 2024 Jun 11;7(6):e2414354. doi: 10.1001/jamanetworkopen.2024.14354 (PMC11167499; doi:10.1001/jamanetworkopen.2024.14354)
Supplement: Supplement 1. — eTable 1. International Classification of Diseases Tenth Revision (ICD-10) Codes Used to Define the Cohort eTable 2. International Classification of Diseases Tenth Revision (ICD-10) Codes Used to Identify Arterial Grafting eTable 3. Definition of Hospital Quality Based Only on Risk-Adjusted Male Mortality eTable 4. Definition of Hospital Quality With Risk-Adjustment Controlling for Percentage of Female Patients at the Hospital Level eFigure. Conceptual Model Evaluating Factors That Contribute to Females’ Higher Mortality After Coronary Artery Bypass Grafting [file jamanetwopen-e2414354-s001.pdf]

Supplementary Online Content

Wagner CM, Ibrahim AM. Sex disparities after coronary artery bypass grafting and hospital quality. *JAMA Netw Open*. 2024;7(6):e2414354. doi:10.1001/jamanetworkopen.2024.14354

eTable 1 International Classification of Diseases Tenth Revision (ICD-10) Codes Used to Define the Cohort
eTable 2. International Classification of Diseases Tenth Revision (ICD-10) Codes Used to Identify Arterial Grafting
eTable 3. Definition of Hospital Quality Based Only on Risk-Adjusted Male Mortality
eTable 4. Definition of Hospital Quality With Risk-Adjustment Controlling for Percentage of Female Patients at the Hospital Level
eFigure. Conceptual Model Evaluating Factors That Contribute to Females' Higher Mortality After Coronary Artery Bypass Grafting

This supplementary material has been provided by the authors to give readers additional information about their work.

© 2024 Wagner CM et al. *JAMA Network Open.*

eTable 1. International Classification of Diseases Tenth Revision (ICD-10) codes used to define the cohort

**Procedure Code**	02100Z8	021108W	02114A9	02120Z8	02130AW
0210083	02100Z9	0211093	02114AW	02120Z9	02130J9
0210089	02100ZC	0211098	02114K9	02120ZC	02130K3
021008C	02100ZF	0211099	02114KW	02120ZF	02130K9
021008W	0210488	021109C	02114Z3	0212493	02130KW
0210093	0210489	021109F	02114Z8	0212498	02130Z3
0210098	0210493	021109W	02114Z9	0212499	02130Z8
0210099	0210498	02110A3	0212089	021249W	02130Z9
021009C	0210499	02110A8	021208W	02124A9	02130ZC
021009F	021049W	02110A9	0212093	02124AW	02130ZF
021009W	02104A3	02110AC	0212098	02124K3	0213483
02100A3	02104A8	02110AF	0212099	02124KW	0213493
02100A8	02104A9	02110AW	021209C	02124Z3	0213499
02100A9	02104AC	02110J9	021209F	02124Z9	021349W
02100AC	02104AF	02110JW	021209W	02124ZF	02134A3
02100AF	02104AW	02110K3	02120A3	0213083	02134K3
02100AW	02104J3	02110K8	02120A8	0213089	02134K9
02100J3	02104K3	02110K9	02120A9	021308W	02134Z3
02100J8	02104K9	02110KC	02120AC	0213093	02134Z8
02100J9	02104KW	02110KW	02120AF	0213098	02134Z9
02100JF	02104Z3	02110Z3	02120AW	0213099	
02100JW	02104Z8	02110Z8	02120J3	021309C	
02100K3	02104Z9	02110Z9	02120J9	021309F	
02100K8	02104ZC	02110ZC	02120JW	021309W	
02100K9	02104ZF	02110ZF	02120K3	02130A3	
02100KC	0211083	0211493	02120K9	02130A8	
02100KW	0211088	0211499	02120KW	02130A9	
02100Z3	0211089	021149W	02120Z3	02130AC	

eTable 2. International Classification of Diseases Tenth Revision (ICD-10) codes used to identify arterial grafting

Procedure Code	02120A8
02100AW	02120A9
0210088	02120AC
02104AW	02124A8
02110AW	02124A9
021114W	02124AC
02120AW	02130A8
02124AW	02130A9
02130AW	02130AC
02134AW	02134A8
02100A8	02134A9
02100A9	02134AC
02100AC	02100AF
02104A8	02104AF
02104A9	02110AF
02104AC	02114AF
02110A8	02120AF
02110A9	02124AF
02110AC	02130AF
02114A8	02134AF
02114A9	02100A3
02114AC	02104A3
02114A3	02110A3
02120A3	02124A3
02130A3	02134A3
02104Z3	02104Z8
02104Z9	02104ZC
02104ZF	02110Z8
02110Z9	02110ZC
02100Z8	02100Z9
02110ZC	02120Z8
02120Z9	02130Z8
02130Z9	02130ZC

© 2024 Wagner CM et al. *JAMA Network Open.*

eTable 3. Definition of hospital quality based only on risk-adjusted male mortality

	Percentage of females	Absolute difference in risk-adjusted mortality *(percentage points)*
**Highest quality**	27.2%	1.24 (1.19-1.28)
**High quality**	26.9%	1.37 (1.33-1.42)
**Average quality**	26.6%	1.53 (1.47-1.59)
**Low quality**	26.7%	1.59 (1.52-1.65)
**Lowest quality**	28.0%	1.76 (1.68-1.84)

© 2024 Wagner CM et al. *JAMA Network Open.*

eTable 4. Definition of hospital quality with risk-adjustment controlling for percentage of female patients at the hospital level

	Percentage of females	Absolute difference in risk-adjusted mortality *(percentage points)*
**Highest quality**	24.5%	1.14 (1.10-1.18)
**High quality**	25.6%	1.29 (1.24-1.33)
**Average quality**	27.0%	1.46 (1.41-1.52)
**Low quality**	28.3%	1.53 (1.47-1.60)
**Lowest quality**	30.0%	1.66 (1.57-1.75)

© 2024 Wagner CM et al. *JAMA Network Open.*

eFigure. Conceptual model evaluating factors that contribute to females' higher mortality after coronary artery bypass grafting

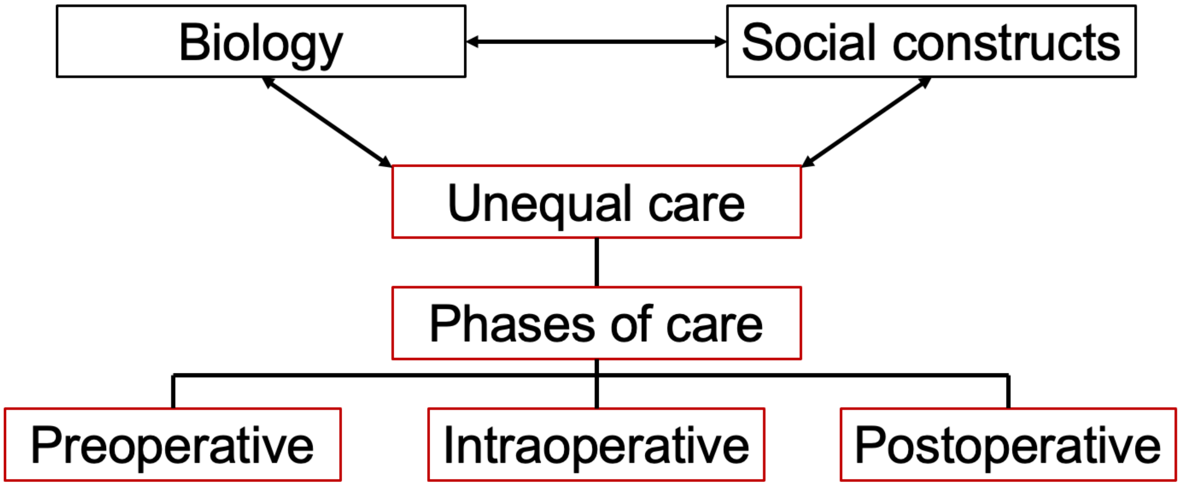

A complex interaction between biological differences, social constructs (i.e., the way males and females interact differently in society), and unequal care likely contribute to the sex disparity after coronary artery bypass grafting.

© 2024 Wagner CM et al. *JAMA Network Open.*
