# Supplementary material for: Sex Disparities After Coronary Artery Bypass Grafting and Hospital Quality
Source: JAMA Netw Open. 2024 Jun 11;7(6):e2414354. doi: 10.1001/jamanetworkopen.2024.14354 (PMC11167499; doi:10.1001/jamanetworkopen.2024.14354)
Supplement: Supplement 2. — Data Sharing Statement [file jamanetwopen-e2414354-s002.pdf]

Data Sharing Statement

Wagner. Sex Disparities After Coronary Artery Bypass Grafting and Hospital Quality. *JAMA Netw Open*. Published June 11, 2024. doi:10.1001/jamanetworkopen.2024.14354

Data
Data available: No

Additional Information
Explanation for why data not available: Due to data use agreements and the sensitive nature of claims data, data will not be available to others.
